# High-Pitch Coronary CT Angiography at 70 kVp With Low Contrast Medium Volume: Comparison of 80 and 100 kVp High-Pitch Protocols

**DOI:** 10.1097/MD.0000000000000092

**Published:** 2014-11-07

**Authors:** Long Jiang Zhang, Li Qi, Carlo N. De Cecco, Chang Sheng Zhou, James V. Spearman, U. Joseph Schoepf, Guang Ming Lu

**Affiliations:** Department of Medical Imaging, *Jinling Hospital*, Medical School of *Nanjing* University, *Nanjing*, *China*.

## Abstract

The purpose of this article is to evaluate image quality and radiation dose of prospectively electrocardiogram (ECG)-triggered high-pitch coronary computed tomography angiography (CCTA) at 70 kVp and 30 mL contrast medium.

One hundred fifty patients with a heart rate ≤70 beats per minute (bpm) underwent CCTA using a second-generation dual-source computed tomography (CT) scanner and were randomized into 3 groups according to tube voltage and contrast medium volume (370 mg/mL iodine concentration) (100 kVp group, 100 kVp/60 mL, n = 55; 80 kVp group, 80 kVp/60 mL, n = 44; 70 kVp group, 70 kVp/30 mL, n = 51). Objective and subjective image quality along with the effect of heart rate (HR) and body mass index (BMI) was evaluated and compared between the groups. Radiation dose was estimated for each patient.

CT attenuation and image noise were higher in the 80 and 70 kVp groups than in the 100 kVp group (all *P *< 0.001). Signal-to-noise ratios (SNRs) and contrast-to-noise ratios (CNRs) were lower in the 70 kVp group than in the 80 and 100 kVp groups (all *P *< 0.05). There was no difference for subjective image quality between the groups (*P *> 0.05). HR did not affect subjective image quality (all *P *> 0.05), while patients with BMI <23 kg/m^2^ had higher image quality than patients with BMI ≥23 kg/m^2^ (*P *< 0.05). Compared with the 100 kVp group, the radiation dose of the 70 kVp group was reduced by 75%.

In conclusion, prospectively ECG-triggered high-pitch 70 kVp/30 mL CCTA can obtain diagnostic image quality with lower radiation dose in selected patients with BMI <23 kg/m^2^ compared with 80/100 kVp/60 mL CCTA.

## INTRODUCTION

Coronary computed tomography angiography (CCTA) has emerged in recent years as a rapid and reliable technique for the diagnosis of coronary artery disease, demonstrating a high sensitivity and negative predictive value.^1,2^ Large multicenter clinical trials have supported the use of CCTA in the emergency department for patients with suspected acute coronary artery syndrome.^3,4^ In light of increasing use, efforts to reduce radiation dose associated with CCTA continue to be desirable.^5^ Several radiation dose-reduction measures have been developed, including low tube voltage, low tube current, prospective electrocardiogram (ECG) triggering, high-pitch acquisition, iterative reconstruction, ECG pulsing, automated kV adaption, and tube filtering.^6,7^ These dose-saving techniques have been individually or jointly used to require the least radiation dose possible while maintaining diagnostic image quality.

Lowering tube voltage is one of the most effective dose (ED)-reduction techniques as it can not only reduce radiation dose but also lower iodinated contrast medium requirements while maintaining diagnostic image quality, especially when combined with iterative reconstruction algorithms.^6–8^ Currently, low tube voltage, such as 100 kVp, has been recommended for performing CCTA in patients with body weight <90 kg.^9^ Some reports have indicated that diagnostic image quality for CCTA with lower radiation dose can be obtained using a tube voltage of 80 kVp.^7,10,11^ Combining other dose-saving techniques, such as high-pitch acquisition or reduced tube current, can substantially reduce radiation dose compared with standard CCTA protocols.^12,13^ Recently, 70-kVp tube voltage has been made available for the clinical setting. Gnannt et al^14^ demonstrated the feasibility of 70-kVp CT imaging in neck studies. Duan et al^15^ found that 70-kVp CT angiography (CTA) of peripheral arteries had a sensitivity of 100%, specificity of 93.5%, and accuracy of 96.1% in diagnosing peripheral arterial occlusive diseases using conventional angiography as the reference standard. They concluded that 70 kVp CTA of peripheral arteries can serve as an effective diagnostic tool for the assessment of peripheral arterial diseases. More recently, a tube voltage of 70 kVp has been described to successfully image the coronary arteries.^16–18^ However, to the best of our knowledge, there are no reports to compare high-pitch CCTA on 70, 80, and 100-kVp tube voltage performed with 2nd-generation dual-source CT. This approach could be very useful in selecting optimal CT protocols for maintaining diagnostic image quality and reducing the radiation dose delivered to both the patient and the contrast medium volume.

Thus, the purpose of this study was to evaluate image quality, radiation dose, and potential factors affecting prospectively ECG-triggered high-pitch CCTA at 70 kVp using 30 mL of contrast medium compared with 80 and 100 kVp high-pitch protocols with 60 mL contrast medium volume.

## MATERIALS AND METHODS

### Study Subjects

This prospective study was approved by the local institutional review board and all patients gave written informed consent. One hundred sixty-two consecutive patients with suspected coronary artery disease were enrolled in this prospective study from September to December 2013. Inclusion criteria were age >18 years, body mass index (BMI) ≤25 kg/m^2^, and heart rate (HR) ≤70 beats per minute (bpm). Exclusion criteria included prior reactions to iodinated contrast medium, impaired renal function (creatinine level ≥120 μmol/L), hemodynamic instability, pregnancy, and prior revascularization with stents or bypass surgery. Patients with any heart rhythm other than sinus rhythm or a HR variability of >30 bpm were also excluded.

All patients received nitroglycerin (0.1 mg per dose; Nitroglycerin Inhaler; Jingwei Pharmacy Co, Ltd, Jinan, China) sublingually 5 minutes before the CCTA scan. In the absence of contraindications, patients with a HR >70 bpm received a tablet of 25 mg metoprolol tartrate (AstraZeneca Pharma, Wuxi, Jiangsu, China) 1 hour before the CCTA examination.

### CT Parameters and Image Reconstruction

All CT examinations were performed on a dual-source CT scanner (Somatom Flash; Siemens Medical Solutions, Forchheim, Germany) equipped with an integrated circuit detector.^19^ The patients were randomized into 3 groups according to the tube voltage setting and contrast medium volume. The 100-kVp group patients received 60 mL iodinated contrast medium (iopromide, Ultravist 370 mg I/mL; Bayer Schering Pharma, Berlin, Germany); the 80-kVp group patients received 60 mL iodinated contrast medium (iopromide, Ultravist 370 mg I/mL; Bayer Schering Pharma); and the 70-kVp group patients received 30 mL iodinated contrast medium (iopromide, Ultravist 370 mg I/mL; Bayer Schering Pharma). All patients underwent CCTA using prospectively ECG-triggered high-pitch (3.4) spiral acquisitions. Acquisition parameters were as follows: detector collimation, 2 × 64 × 0.6 mm; gantry rotation time, 280 millisecond; and effective tube current–time product, 320 mAs per rotation. For all studies, automated tube current modulation (CAREDose 4D; Siemens, Forchheim, Germany) was enabled. Image acquisition was prospectively triggered by the patient’s ECG at 60% of the R–R′ interval. The contrast agent was injected into an antecubital vein through an 18-gauge catheter with a flow rate of 4 mL/second followed by 40 mL of saline solution at the same rate. Contrast agent application was controlled by bolus-tracking technique. A region of interest (ROI) was placed into the aortic root, and image acquisition started 4 seconds after the signal attenuation reached the predefined threshold of 100 Hounsfield units (HU). Images were reconstructed with a section thickness of 0.75 mm, a reconstruction increment of 0.5 mm, and a medium soft tissue convolution kernel (I26f), using sinogram-affirmed iterative reconstruction (SAFIRE; Siemens) at a strength level of 3 for all CCTA studies. All reconstructed images were transferred to a dedicated workstation (3D Workplace, Siemens) equipped with cardiac post-processing software (SyngoVia CT Coronary, Siemens). Image post-processing techniques included curved multiplanar reformation and volume-rendered displays.

### Objective Analysis of Image Quality

To evaluate objective parameters of image quality, 1 observer measured CT attenuation and image noise of the ascending aorta, the proximal segments of the right coronary artery (RCA), left main artery (LM), left anterior descending artery (LAD), left circumflex artery (LCX), and the adjacent pericardial fat in each patient. A circular ROI was placed in the lumen of the target vessel. The largest possible ROI size was chosen without including parts of the vessel wall, calcification, or plaques. Image noise was determined by measuring the standard deviation (SD) of CT attenuation in the circular ROI in the target vessel. Signal-to-noise ratio (SNR) and contrast-to-noise ratio (CNR) were calculated. SNR was calculated as the quotient of mean attenuation of the coronary artery and image noise. CNR was determined by dividing contrast attenuation by the image noise of the adjacent pericardial fat tissue.^6^

### Subjective Analysis of Image Quality

**FIGURE 1:**
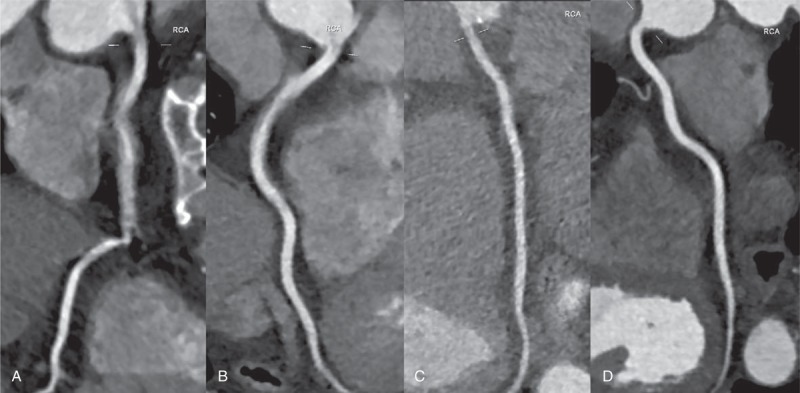
Image examples illustrating the four-point rating scale for image quality. Shown are automatically generated curved multiplanar reformations along the vessel center line of coronary arteries studied with CCTA. Panel A shows a 100 kVp CCTA study which was given a score of 1 because of marked motion artifact in segment 2. Panel B shows a 100 kVp CCTA study which was given a score of 2 because of mild motion artifact in segment 2. Panel C shows a 70 kVp CCTA study which was given a score of 3 because of a mild irregular contour of segment 2. Panel D shows an 80 kVp CCTA study which was given a score of 4.

The American Heart Association coronary artery segmental model^20^ was used for subjective analysis of CCTA image quality. All segments with a diameter of <1.5 mm at their origin were excluded. For avoiding potential bias, observers were blinded to patients’ information and acquisition parameters during image analysis. Two observers rated the image quality of each coronary segment independently by using a 4-point scale (Figure 1), where 4 = excellent, no significant artifact; 3 = good, mild artifact; 2 = acceptable, moderate artifact present but images are still interpretable; and 1 = not evaluable, with severe artifacts rendering diagnostic interpretation impossible.^21^ In the case of disagreement between observers, consensus was reached in a joint reading to determine the final image quality score per segment. A per-vessel and per-patient image quality score was defined as the lowest score found in any segment for each vessel or each patient.

### Estimation of Radiation Dose

The volumetric CT dose index and dose-length product (DLP) were recorded for each examination according to the patient’s study protocol report. EDs were estimated by multiplying the DLP reported by the scanner by a conversion factor of 0.014 mSv/mGy·cm according to standard methodology outlined in the most recent guidelines.^9,22^ Additionally, the size-specific dose estimate was calculated on the basis of each patient’s effective diameter (or body size) as measured from the scout images or axial images covering the thorax, which were obtained following magnifying the field of view of CCTA scanning.^23,24^

### Statistical Analysis

Statistical analyses were performed using SPSS version 16.0 (SPSS Inc, Chicago, IL). Quantitative variables were expressed as mean values ± SD, while categorical variables were expressed as frequencies or percentages. The Kolmogorov–Smirnov test was used to test whether data was normally distributed. Analysis of variance tests were used to analyze age, HR, BMI, CT attenuation, noise, image quality scores, CNR, SNR, and radiation dose parameters among the 3 groups. Chi-square test was used to compare the difference of sex and other clinical variables among the 3 groups. An independent sample Student *t* test was used to compare image quality score between the patients with HR ≥65 bpm and those with HR <65 bpm in 3 different groups. An independent sample Student *t* test was also used to compare image quality scores between the patients with BMI ≥23 kg/m^2^ and those with BMI <23 kg/m^2^ in the 3 groups. Interobserver variability between the 2 readers with regard to subjective image quality assessment was evaluated with κ statistics. A κ value of <0.20 indicated poor agreement; a κ value of 0.21–0.40, fair agreement; a κ value of 0.41–0.60, moderate agreement; a κ value of 0.61–0.80, good agreement; and a κ value of 0.81–1.00, very good agreement. The relationship between HR, BMI, and image quality was evaluated with partial correlation analysis. *P* values <0.05 were regarded as significant.

## RESULTS

### Study Population

Among 38 patients with HR >70 bpm before treatment, metroprolol tartare successfully decreased the HR <70 bpm, resulting in 150 patients available for CCTA. The 100, 80, and 70 kVp groups included 55, 44, and 51 patients, respectively. Table 1 shows the patients’ demographics. There were no differences for patients’ age, sex, and BMI among the 3 groups (all *P *> 0.05).

**TABLE 1:**
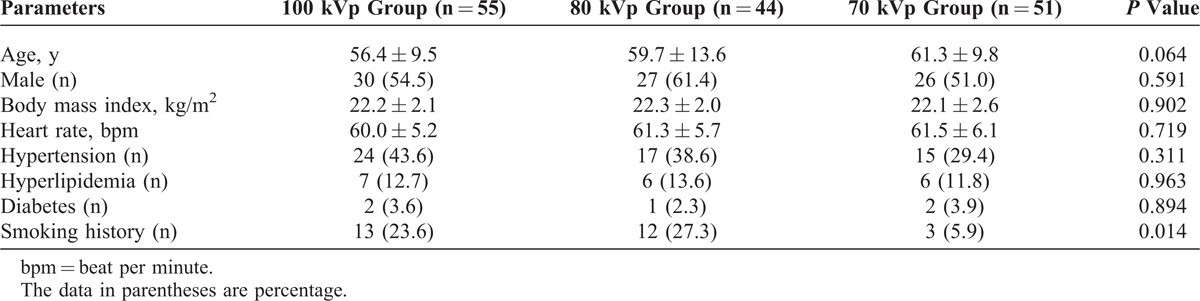
Patient Demographics

### Objective Analysis of Image Quality

Table 2 shows quantitative measurements in different target vessel locations among the 3 groups. Mean CT attenuation in all the target vessel locations were higher in the 70 and 80 kVp groups than those in the 100 kVp group (both *P *< 0.001). However, image noise in the 70 and 80 kVp groups was higher than that in the 100 kVp group (both *P *< 0.001). The SNR in the 70 kVp group was lower than in the 80 and 100 kVp groups (both *P *< 0.05), while no difference was found for the SNR between the 80 and 100 kVp groups (*P *> 0.05). Lower CNRs of the RCA, LAD, and LCX were found in the 70 kVp group than in the 80 and 100 kVp groups (*P *< 0.05), while no significant difference for the CNR was identified between the 80 and 100 kVp groups (*P *> 0.05). No difference in the CNR was found for the aorta or LM among the 3 groups (all *P *> 0.05).

**TABLE 2:**
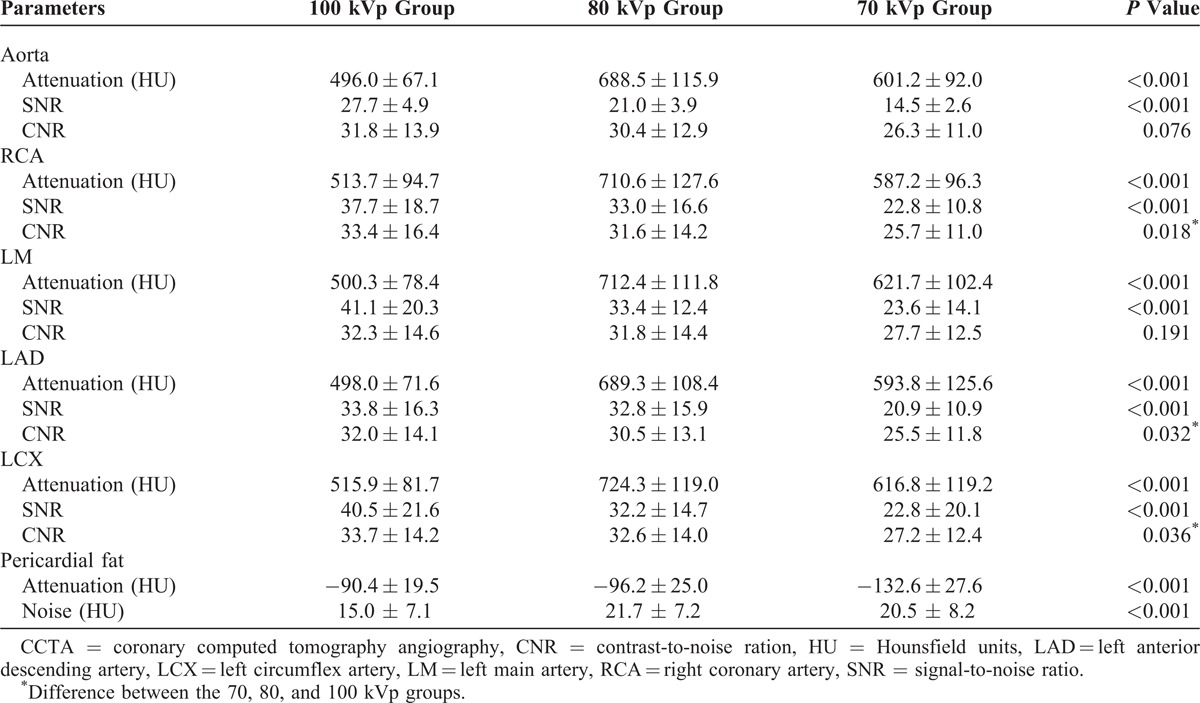
Objective Image Quality Evaluation of CCTA Among 3 Groups

### Subjective Analysis of Image Quality

**FIGURE 2:**
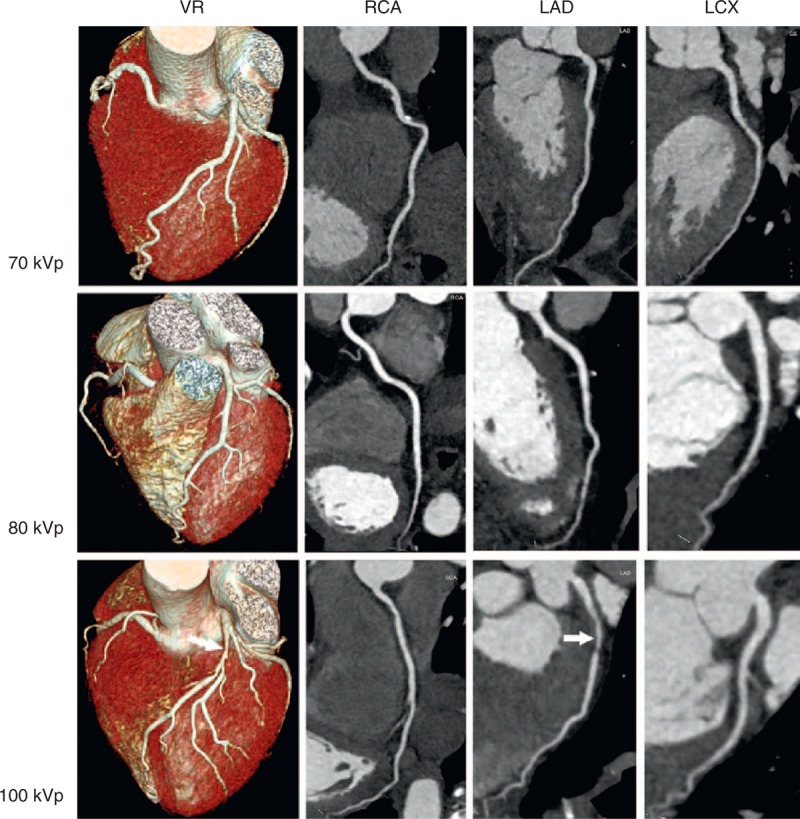
Representative CCTA images in three different patients investigated with 70 kVp, 80 kVp, and 100 kVp. All CCTA images were assigned a score of 4 by the two readers. Note severe stenosis (arrows) in the proximal segment of the LAD of the subject in the 100 kVp group. VR = volume rendering; RCA = right coronary artery; LAD = left descending coronary artery; LCX = left circumflex coronary artery.

For the 150 patients, 390 segments were not assessed because of anatomic variants (n = 22 for the 100 kVp group, n = 14 for the 80 kVp group, and n = 10 for the 70 kVp group) or a caliber of <1.5 mm (n = 116 for the 100 kVp group, n = 127 for the 80 kVp group, and n = 131 for the 70 kVp group). In total, 1860 segments were available for image analysis. In the 100 kVp group (n = 687 segments), 15 segments (2%) were rated as a score of 1, 24 segments (3%) as score of 2, 168 segments (25%) as score of 3, and 480 segments (70%) as score of 4. In the 80 kVp group (n = 549 segments), 20 segments (4%) were rated as score 1, 27 segments (5%) as score 2, 177 segments (32%) as score 3, and 324 segments (59%) as score 4. In the 70 kVp group (n = 624 segments), 8 segments (1%) were rated as score 1, 41 segments (7%) as score 2, 200 segments (32%) as score 3, and 375 segments (60%) as score 4. Among 150 patients, 18 patients (12%, n = 8 [15%] for the 100 kVp group, n = 6 [14%] for the 80 kVp group, and n = 4 [8%] for the 70 kVp group) had nondiagnostic segments on CCTA. The segments with nondiagnostic image quality were located in the RCA (n = 16) and LCX (n = 27), and were considered to result from coronary motion artifact. Figure 2 illustrates representative CCTA studies with excellent image quality from 3 different patients in the 70, 80, and 100 kVp groups.

Table 3 shows the results of subjective evaluation of image quality among the 3 groups. On a per-patient basis, there was no difference for image quality (all *P *> 0.05). However, we did find lower image quality of the LAD and LCX in the 70 kVp group compared to the 80 and 100 kVp groups (both *P *< 0.05). Interobserver agreement was good to moderate for the 100 kVp group (κ = 0.68), the 80 kVp group (κ = 0.41), and the 70 kVp group (κ = 0.58).

**TABLE 3:**
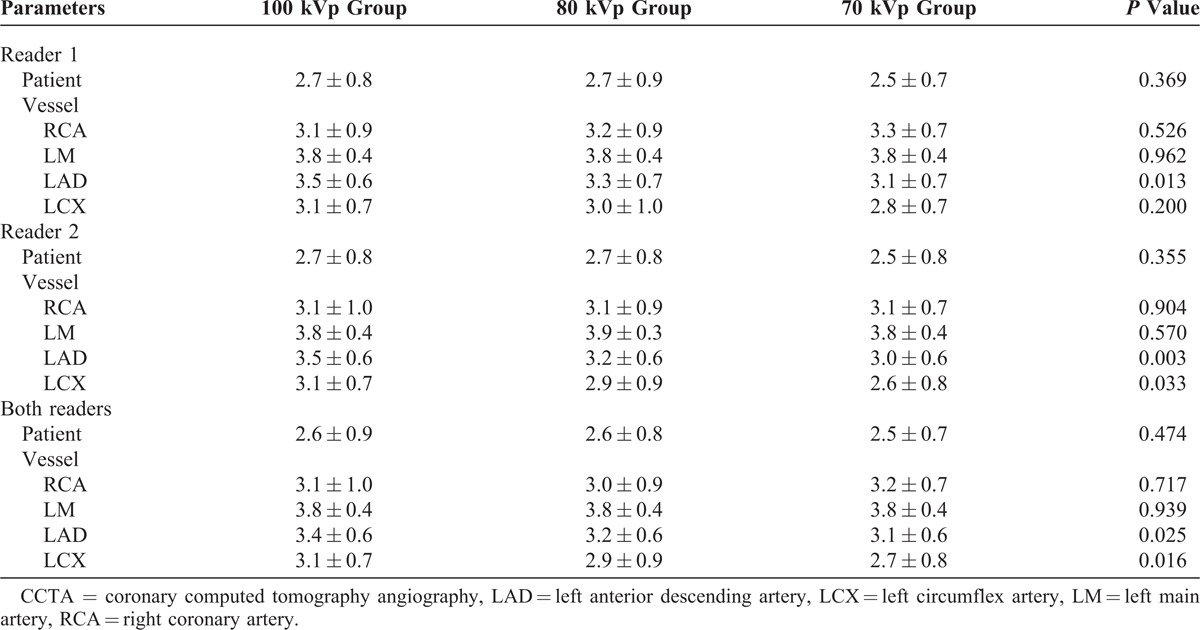
Subjective Image Quality Evaluation of CCTA Among 3 Groups

### Effect of HR and BMI on Image Quality

We further analyzed the effect of HR and BMI on the objective and subjective image quality evaluation. For objective image quality evaluation, no difference was found for all measurements between the patients with HR <65 bpm and HR ≥65 bpm in all the three groups (all *P *> 0.05) except for mean CT attenuation of the RCA (*P* = 0.034) in the 80 kVp group (Supplementary Table 1, http://links.lww.com/MD/A69). However, we found that BMI had an effect on objective image quality (Supplementary Table 2, http://links.lww.com/MD/A70). Especially, in the 70 kVp group, SNR of the aorta, CNR of the LAD, and mean CT attenuation of the RCA and LAD were higher in patients with BMI <23 kg/m^2^ compared to patients with BMI ≥23 kg/m^2^ (all *P *< 0.05), while there was no difference for any other measurements between the 2 subgroups (all *P *> 0.05).

For subjective image quality evaluation, we did not find any effect related to HR on overall image quality on a per-patient basis (all *P *> 0.05) (Supplementary Table 3, http://links.lww.com/MD/A71). For BMI, we found that patients with BMI ≥23 kg/m^2^ had lower image quality than patients with BMI <23 kg/m^2^ in the 70 kVp group on a per-patient basis, especially in the LCX (*P* = 0.035) (Supplementary Table 4, http://links.lww.com/MD/A72).

Partial correlation analysis showed poor negative correlation between image quality and BMI (*r* = −0.180, *P* = 0.028) and between overall image quality and HR (*r* = −0.295, *P *< 0.001) for all cases. For the 80 and 100 kVp group, poor negative correlation between image quality and HR (*r* = −0.301, *P* = 0.047; *r* = −0.327, *P* = 0.015; respectively) was found. No correlation was found between BMI and image quality in the 80 and 100 kVp groups (both *P *> 0.05). In the 70 kVp group, no correlation was found between image quality, BMI, and HR (both *P *> 0.05).

### Estimation of Radiation Dose

Table 4 shows the radiation dose estimation resulting from the 3 CCTA protocols. The lowest radiation dose was observed in the 70 kVp group, followed by the 80 kVp group, and the 100 kVp group (both *P *< 0.001). Compared with the 80 and 100 kVp groups, the radiation doses of the 70 kVp group were lower by 56% and 75%, respectively.

**TABLE 4:**
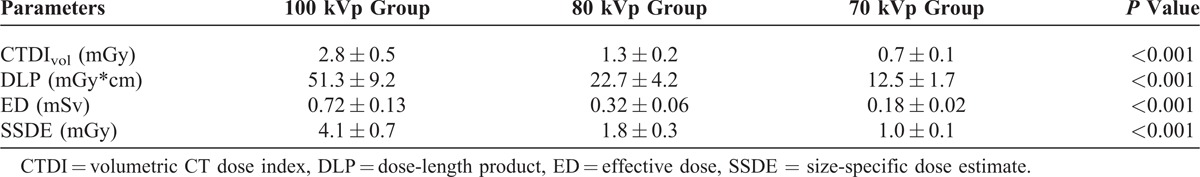
Radiation Dose Estimation Among 3 CCTA Protocols

## DISCUSSION

Our results confirm the potential of using prospectively ECG-triggered high-pitch spiral CCTA at 70 kVp with low contrast volume in a selected population without any detriment in diagnostic image quality compared with the 80 and 100 kVp protocols at standard contrast medium volume. Low tube voltage has been shown to be an effective technique for radiation dose reduction in CT studies with the additional advantage of reduction of contrast medium volume.

Our study showed higher CT attenuation in all measured locations in the 70 and 80 kVp groups than in the 100 kVp group; however, lower SNR and CNR were noted in the 70 kVp group compared to the 80 and 100 kVp groups because of higher noise level in the 70 kVp group. We used the SAFIRE algorithm for all patients in all the 3 groups, which possibly explained the presence of statistical differences in SNR and CNR. Additionally, SAFIRE at a strength level of 3 was arbitrarily used in the 70 kVp studies because it has been successfully applied in the 80 and 100 kVp protocols previously.^8,12^

The optimal arterial contrast attenuation in the coronary arteries at CCTA studies range between 250 and 350 HU.^25–27^ In our study, the mean CT attenuation of all target vessels in the 70 and 80 kVp groups were higher than 550 HU, indicating the possibility of further reductions in contrast medium volume at CCTA, as also recently reported in a CCTA study using 80 kVp tube voltage.^25^ Low tube voltage increases the attenuation of iodinated contrast agents because the mean photon energy of the conventional beam is closer to the maximum absorption of the k-edge of iodine at 33.2 keV.^15^ Thus, with low tube voltage techniques, it is possible to reduce contrast medium volumes while maintaining the same attenuation within target vessels.

Although SNR and CNR were decreased, subjective overall image quality at 70 kVp did not show a significant deterioration in comparison with the 80 and 100 kVp groups. Moreover, in a subgroup analysis, we found lower image quality in the LAD and LCX in the 70 kVp group, which could be related to the higher noise secondary to the lower X-ray beam penetration capability at lower kVp.

Using the high-pitch modality and iterative reconstruction, it was possible to restrict the ED to <1 mSv in all the 3 protocols. However, compared with 100 and 80 kVp acquisitions, the 70 kVp protocol allowed a substantial reduction in radiation dose (75% and 43.8% reduction, respectively) and contrast medium volume (50% reduction), without any significant deterioration in the image quality that was still rated diagnostic for all examinations. The finding of substantial radiation dose reduction in this study is in line with the previous CCTA studies at 70 kVp using a 2nd and 3rd-generation dual-source CT system.^16,18^

In CCTA studies, image quality can be affected by several factors including patient-specific characteristics such as HR and BMI. For this reason, in our study, we evaluated the effect of these 2 parameters on image quality. It has been demonstrated that HR is an important limiting factor in high-pitch CCTA studies.^12,13^ The study by Neefjes et al^13^ indicates that a high-pitch CCTA protocol should be successfully applied in patients with regular and low (<55 bpm) HRs. Other studies restricted the use of the high-pitch CCTA protocol to HRs <65 bpm.^28–31^ In our study, no significant difference was found for subjective image quality between patients with HR <65 bpm and with HR ≥65 bpm; however, a significant difference was found in a per-vessel basis between the 3 groups. In particular, all the nondiagnostic segments were found in the RCA and LCX in subjects with a HR ≥65 bpm. This observation could be related to the fact that the RCA and LCX demonstrate faster movement and larger excursion compared to the LAD and are thus more susceptible to motion artifacts.^32,33^ This result supports the recommendation to limit the use of high-pitch CCTA studies to patients with a HR <65 bpm in order to reduce the number of possible artifacts.

Patient BMI can significantly affect CCTA image quality as well, especially when a low tube voltage is applied. For example, current Society of Cardiovascular Computed Tomography guidelines^9^ recommend acquisition at 100 kVp for patients with BMI ≤30 kg/m^2^ or body weight ≤90 kg. As expected, in our study, we found a significant impact of the BMI on image quality at 70 kVp. In particular, we observed a significant reduction in image quality in studies of patients with a BMI >23 kg/m^2^.

Considering our results regarding the influence of HR and BMI on image quality, it appears recommendable to limit the application of 70 kVp high-pitch CCTA to patients with a BMI <23 kg/m^2^ and a HR <65 bpm in order to obtain diagnostic image quality with low contrast medium volume. For patients with BMI ranging from 23 to 25 kg/m^2^, 80 kVp CCTA appears recommendable to obtain diagnostic examinations. The 100 kVp protocol can be reserved to those patients with a BMI >25 kg/m^2^.

Our findings need to be evaluated in light of the following limitations. First, we only included patients with a BMI <25 kg/m^2^ and a HR <70 bpm in sinus rhythm. Additionally, we studied an East Asian population, who generally has slimmer body types than the average Western population. Thus, our protocol can likely not be generalized for indiscriminate use in less-strictly selected patient populations. Second, we did not investigate the diagnostic accuracy of 70-kVp high-pitch CCTA with invasive coronary angiography as an outside reference standard. However, a recent study by Lee et al^34^ showed, based on a phantom study, that tube voltage does not affect the accuracy of stenosis measurement. Third, patients with stents or prior bypass surgery were excluded in this study; thus, our findings cannot be extrapolated to these populations. Last, the results of our study apply only to a 2nd-generation dual-source CT system and cannot be generalized to other CT platforms.

In conclusion, our study shows that prospectively ECG-triggered high-pitch CCTA at 70 kVp with 30 mL iodinated contrast medium volume can obtain diagnostic image quality with substantially reduced radiation dose in selected patients with BMI <23 kg/m^2^ and HR <65 bpm compared with 80 and 100 kVp CCTA using 60 mL contrast medium volume.
